# Improvements in sleep indices during exam stress due to consumption of a *Bifidobacterium longum*

**DOI:** 10.1016/j.bbih.2020.100174

**Published:** 2020-11-13

**Authors:** Gerard M. Moloney, Caitriona M. Long-Smith, Amy Murphy, Danielle Dorland, Sara Firuzeh Hojabri, Loreto Olavarría Ramirez, David Campos Marin, Thomaz F.S. Bastiaanssen, Anne-Marie Cusack, Kirsten Berding, Fiona Fouhy, Andrew P. Allen, Catherine Stanton, Gerard Clarke, Timothy G. Dinan, John F. Cryan

**Affiliations:** a APC Microbiome Ireland, University College Cork, Cork, Ireland; b Department of Anatomy and Neuroscience, University College Cork, Cork, Ireland; c Teagasc Food Research Centre, Moorepark, Fermoy, Cork, Ireland; d Department of Psychiatry and Neurobehavioural Science, University College Cork, Cork, Ireland

**Keywords:** Psychobiotics, Stress, Sleep, Placebo-controlled, Bif, Bifidobacterium, CANTAB, Cambridge Neuropsychological Test Automated Battery, HPA, hypothalamic-pituitary-adrenal axis

## Abstract

Targeting the gut microbiome as an effective therapeutic strategy for psychological disorders has shown promise in recent years. Variation in the composition of the microbiota and restoration of a stable microbiome using targeted interventions (psychobiotics) including Bifidobacteria have shown promise in pre-clinical studies, but more human data is required on the potential health benefits of these live microorganisms*.* Bifidobacterium including *Bif. longum* 1714 has been shown to dampen the effects of acute stress in humans. However, its effects over a period of prolonged stress have not been examined. A randomised, placebo-controlled, repeated measures, cross-over intervention study was conducted to examine the effects of a probiotic intervention on measures of stress, cognitive performance, and mood in healthy human volunteers. Twenty male students participated in this crossover study. Post-intervention assessments took place during the university exam period, which was used as a naturalistic chronic stressor. Self-reported measures of stress, depression, sleep quality, physical activity, gastrointestinal symptoms, cognition, and mood were assessed by questionnaire. In addition, tests from the Cambridge Neuropsychological Test Automated Battery (CANTAB) were administered to all participants. Stress and depression scores increased in both placebo and probiotic treated groups during the exam period. While overall sleep quality and duration of sleep improved significantly in the probiotic treated group during exam stress compared with the placebo treated group, *B. longum* 1714, similar to placebo treatment, showed no efficacy in improving measures of working memory, visual memory, sustained attention or perception. Overall, while *B. longum* 1714 shows promise in improving sleep quality and duration, it did not alleviate symptoms of chronic stress, depression, or any measure of cognitive assessment. Thus, further mechanistic studies into the ability of *B. longum* 1714 to modulate sleep during prolonged periods of stress are now warranted.

## Highlights

• Using a repeated measures design ​we selected stress and cognitive tests that would examine memory and sustained attention.
• *Bif. longum* had a positive effect on self-reported sleep quality.
• Our data supports the potential role for probiotics in modulating brain health.

## 1 Introduction

In recent years, preclinical studies have established that the use of probiotics that target the microbiome can influence brain development, function and behaviour (Bercik et al., 2011; Buffington et al., 2016; Desbonnet et al., 2010; Savignac et al., 2014, 2015; Long-Smith et al., 2020; Morais et al., 2020; Teichman et al., 2020). Psychobiotics, defined as bacteria that when ingested in adequate amounts produce a positive mental health benefit (Dinan et al., 2013) are thought to function via the brain-gut-axis, and when administered have the potential to have a considerable impact on stress, anxiety-like and depression-like symptoms (Sarkar et al., 2018). The definition of psychobiotics should be expanded to any exogenous influence whose effect on the brain is bacterially-mediated. Furthermore, psychobiotics have demonstrated efficacy in reducing some physiological outputs of anxiety and depression such as immune function, corticosterone/cortisol, neurotransmitters, and brain-derived neurotrophic factor (BDNF) in animal and human studies (Bercik et al., 2011; Bravo et al., 2011; Buffington et al., 2016; Messaoudi et al., 2011). Though a large proportion of the data regarding the effectiveness of probiotics has been generated in preclinical models, particular probiotic strains have shown potential for symptom improvement in irritable bowel syndrome (IBS), a stress sensitive brain-gut-axis disorder with high rates of psychiatric co-morbidity, altered cognitive ability (Kennedy et al., 2014a), and activation of the hypothalamic-pituitary-adrenal axis (HPA) (Whorwell et al., 2006; Aragon et al., 2010; Kennedy et al., 2014b). Stress is also known to influence the composition of the gut-microbiome, a key component of the microbiota-gut-brain axis (Foster et al., 2017; Cruz-Pereira et al., 2020). Heightened stress and anxiety can have a detrimental effect on the composition of the microbiome and the microbiome is now considered a viable therapeutic target for countering the negative effects of stress. Proof-of-principle studies in healthy human volunteers have demonstrated the efficacy of a number of prebiotics, fermented drinks containing probiotics and combinations of probiotics that are able to alter stress outputs, cognitive performance and self-reported psychological variables (Benton et al., 2007; Chung et al., 2014; Messaoudi et al., 2011; Steenbergen et al., 2015; Allen et al., 2016b; Wang et al., 2019).

Emerging from a preclinical screening platform in mice we were able to identify clinically relevant candidate strains that can selectively impact stress-related behaviours and improve cognitive performance in rodents (Savignac et al., 2014, 2015). We identified *Bifidobacterium longum* 1714 (1714) as a probiotic strain that showed potential to treat stress and anxiety disorders in the clinic. In fact, recent data from our lab showed that *Bif. longum* was efficacious in reducing the effects of acute stress and improving memory in healthy volunteers (Kelly et al., 2017; Wang et al., 2019). Thus, we proposed to assess the value of consuming the strain *Bif. longum*, compared with a placebo, in ameliorating stress measures, in addition to measures of cognitive performance in healthy individuals in response to a naturalistic chronic stressor, university exam stress, using a double-blind, randomised, placebo-controlled, cross-over design. We assessed the self-reported measure of stress as our primary outcome along with self-reported sleep quality, in addition to cognitive performance. Furthermore, we measured the cortisol awakening response, hair cortisol and the composition of the microbiome before and after chronic exam stress.

## 2 Materials and methods

### 2.1 Ethics

The study was approved by the Clinical Research Ethics Committee of the Cork Teaching Hospitals (study number APC080) and conducted in accordance with the ICH Guidelines on Good Clinical Practice and the Declaration of Helsinki. Written informed consent was obtained from all participants at the screening visit, before any study procedures were conducted. Participants were free to withdraw from the study at any time.

### 2.2 Study design

#### 2.2.1 Study participants

**Fig. 1:**
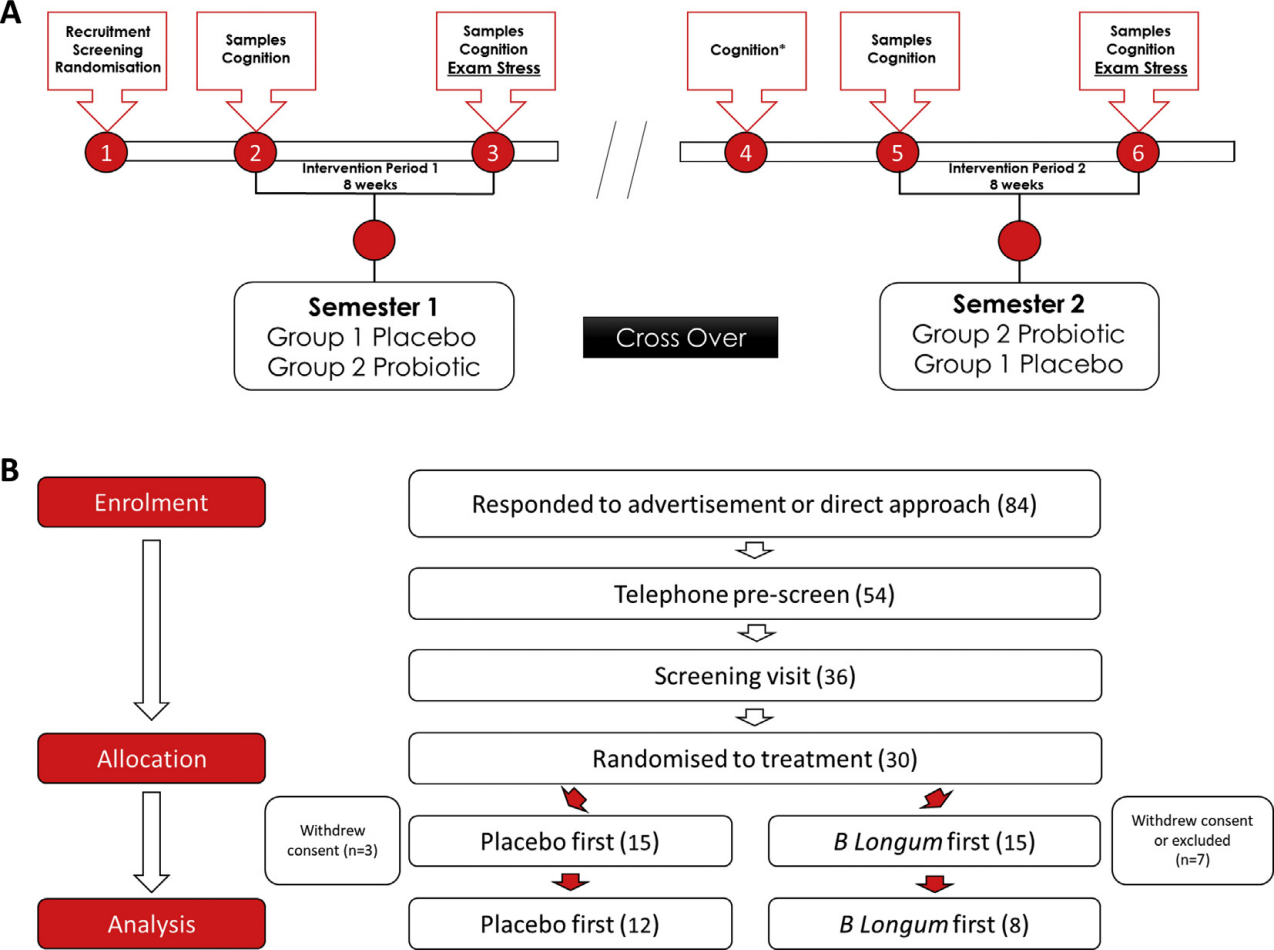
**Overview of Probiotic Intervention in Chronic Stress**. (A) Visit number is denoted by red circles, visit 1, participants gave informed consent and were recruited to the study and randomised to either a placebo or probiotic group. Visit 2, stool, hair, blood and saliva samples were taken before an 8-week intervention period on placebo or probiotic followed by visit 3, the end of semester 1 visit where stool, hair, blood and saliva samples were obtained. N=9 withdrew consent prior to commencing on the intervention product, and n=1 participant withdrew consent during the first intervention phase due to unwillingness to attend further study visits mainly due to scheduling difficulties. All participants switched intervention for semester 2 which commenced with visit 5 where stool, hair, blood and saliva samples were taken before the 2nd 8-week intervention period. Visit 6 took place at the end of the 8-week intervention where once again, stool, hair, blood and saliva samples were taken from each participant. Semester one and two were conducted based on the exam schedule of the volunteers. Full details of each study visit are in Table 2. **(B)** Study recruitment, 84 volunteers responded to advertisement and direct contact; 54 were pre-screened; 36 were invited to a screening visit; and thirty were enrolled in the study and randomised to treatment. Following treatment assignment, 3 withdrew from the placebo group and 7 withdrew from the probiotic group. (For interpretation of the references to colour in this figure legend, the reader is referred to the web version of this article.)

Study participants were recruited via advertisement and direct contact to the student population of University College Cork. Eighty-four volunteers responded to advertisement and direct contact; 54 were pre-screened by telephone call (64%); 36 were invited to a screening visit (43%); and thirty were enrolled in the study and randomised to treatment (36%). Inclusion criteria: participant must be able to give written informed consent; be between 18 and 30 years of age; be male; be in generally good health as determined by the investigator (Fig. 1). **Exclusion criteria** were: being less than 18 and greater than 40 years of age; having a significant acute or chronic illness; having a condition or taking a medication that would interfere with the objectives of the study, pose a safety risk or confound the interpretation of the study results – subjects should have a wash-out period of 4 weeks; current prebiotic or probiotic use – subjects should have a wash-out period of 4 weeks; excessive use of vitamin D supplementation; not being fluent in English; having dyslexia or dyscalculia; being a current or past smoker; being considered to be poor attendees or unlikely for any reason to be able to comply with the trial; using treatment involving experimental drugs – participation in a trial should be completed not less than 30 days prior to this study; and having a malignant disease or any concomitant end-stage organ disease. Prior to testing days, participants were asked to refrain from strenuous exercise and alcohol 24 ​h before the session, and from caffeine 3 ​h prior to the session.

#### 2.2.2 Study design

The study was a double-blind, randomised, placebo-controlled, repeated measures, cross-over design. At the screening visit, two weeks before intervention start, study participants were asked about their demographics, general medical history, medication record, and mode of delivery at birth. Furthermore, the participants were screened using the MINI International Psychiatric Interview (to exclude subjects with a significant DSM-V psychiatric diagnosis) and completed a battery of self-report scales including the Childhood Trauma Questionnaire-Short Form (CTQ-SF), Ten Item Personality Inventory (TIPI), Cambridge Behaviour Scale (CBS), Interpersonal Reactivity Index (IRI), Autism Quotient (AQ), State-Trait Anxiety Inventory (STAI) – trait part, and Ways of Coping Questionnaire (WAYS). Participants whose first language was English completed the National Adult Reading Test-2 (NART-2) to determine IQ levels. Subsequently, the volunteer did a brief practice of the Cambridge Neuropsychological Test Automated Battery ("CANTAB® [Cognitive assessment software]. Cambridge Cognition (2017). www.cantab.com,") in order to mitigate against learning effects on measurements of cognition [the Motor Screening Test (MOT), and to stage 1 of the Paired Associated Learning (PAL) and stage 1 of the Rapid Visual Information Processing (RVP)]. The full study battery included the MOT, Spatial Span (SSP), Emotion Recognition Test (ERT), PAL, and RVP, which were presented using a Latin Square order. All tests were presented on a touch-screen monitor. A test administrator sat with the participant to provide verbal instructions from a standardised script.

Upon enrolment in the study, participants were randomly assigned to either of two groups using block randomisation. One of the groups received placebo (corn starch, magnesium stearate, hypromellose & titanium dioxide) in the first intervention period and probiotic (*B. longum AH1714,* corn starch, magnesium stearate, hypromellose & titanium dioxide, to achieve a target dose of 1 x 10^9^ ​cfu/day) during the second period, while the other group received the opposite, in a cross-over double-blind design (Fig. 1). The intervention period was approximately eight weeks, during the run-up to the first and second semester exam periods in UCC, Cork, dependant on each individual’s exam timetable scheduling. The post-intervention visits took place during the participant’s exams, but not on the day of an exam. The probiotic and placebo were in capsule form and taken once a day. Participants were instructed to consume the product every morning, before, with or after food. They were instructed not to consume the product with fruit juice or warm or hot food and drink, and not to consume such items for at least 15 ​min after ingestion of the product. On the morning of each pre- and post-intervention visit, participants collected four saliva samples (Salivette ®). At each visit, a brief physical examination was carried out to determine body mass index (BMI). Blood, saliva, hair and stool samples were collected. Safety blood profiling (biochemistry and haematology) was performed in a local hospital laboratory. Participants filled in self-report scales and questionnaires, including the Food Frequency Questionnaire (FFQ), International Physical Activity Questionnaire (IPAQ), Gastrointestinal Visual Analogue Scale (GI-VAS), Bristol Stool Chart, Pittsburgh Sleep Quality Index (PSQI), Perceived Stress Scale (PSS), Reading the Mind in the Eyes, and the Beck’s Depression Inventory second edition (BDI-II). Cognitive performance was measured using a battery of tests from the CANTAB suite. At the post-intervention visit, the Primary Appraisal Secondary Appraisal (PASA) was additionally included. At the final visit, participants were asked to rate which exam period they found most stressful and most difficult. Approximately ten weeks before the second exam period, participants had a reminder CANTAB session, the same as that which they had at the screening visit, to mitigate against learning effects and mimic the first phase. At this visit, and all study visits, it was also ensured that participants still met the inclusion and exclusion criteria. For an overview of the timeline, see Fig. 1, Table 2. Adverse events were monitored and recorded throughout the study.

#### 2.2.3 Biological sample collections and analysis

##### 2.2.3.1 Stool collection and storage

Faecal samples from the morning of the visit were collected into plastic containers containing an Anaerogen sachet. Participants were instructed to keep the sample in a cool place until delivery at the visit time.

##### 2.2.3.2 Saliva collection and storage

Saliva samples for the cortisol awakening response (x 8: 2 x mornings x 4 samples/morning) were collected using Salivette devices. Participants were instructed to keep the sample in a cool place until delivery at the visit time and the samples were then stored at −80^O^C.

##### 2.2.3.3 Hair collection and storage

A hair sample of approximately 150 strands (ideally 2–6 ​cm long) was cut close to the scalp from the back of the head in a position deemed least noticeable and most comfortable for the participant. The side closest to the scalp was marked and the sample stored at room temperature for subsequent analysis of chronic cortisol levels.

### 2.3 Sample analysis

#### 2.3.1 Cortisol awakening response

##### 2.3.1.1 Enzyme-linked immunosorbent assay (ELISA) kits

(Enzo Life Sciences) were used to measure cortisol concentrations. Salivary cortisol was analysed using Enzo Life Sciences (Exeter, UK), enzyme-linked immunosorbent assay (ELISA) kits (Catalogue no: ADI-901-071) according to manufacturer’s instructions. Lower limit of detection ​= ​56.72 ​pg/mL, Inter and intra-assay coefficients of variability were 10.5% and 13.5% respectively.

### 2.4 DNA isolation, sequencing and bioinformatic analysis (16S rRNA gene) from faecal samples

Stool samples were collected from each participant at four time points as shown in Fig. 1. DNA was extracted from stool samples using the RBB method (Yu and Morrison, 2004). Briefly, 0.2g faecal sample were weighed and added to 2 ​ml screw-cap tubes containing 0.25 ​g of a 1:1 mix of 0.1 ​mm and 1.5 ​mm diameter sterile zirconia beads plus a single 2.5 ​mm diameter bead. To this, 1 ​ml of lysis buffer was added (500 ​mM NaCl, 50 ​mM tris-HCL, pH 8.0, 50 ​mM EDTA and 4% sodium dodecyl sulphate (SDS)). Each sample was then homogenised using a Mini-Beadbeater™ at maximum speed for 3 ​min and incubated at 70 ​°C for 15 ​min to lyse the cells. Samples were centrifuged for 5 ​min ​at 16,000*×g* and the supernatant was transferred to a fresh Eppendorf tube. The bead beating, heating and centrifugation steps were repeated using 300 ​μl of lysis buffer and the supernatant was pooled. Following this, 260 ​μl of 7.5M ammonium acetate was added, and the samples were vortexed and incubated on ice for 5 ​min. Isopropanol was added to precipitate the DNA and samples were centrifuged to pellet the nucleic acid. The pellets were then washed with 70% ethanol and allowed to dry before being dissolved in 100 ​μl ​TE buffer. The DNA was treated with RNAse and Proteinase K and washed with Qiagen buffers AW1 and AW2 using columns provided in the QIAmp Fast DNA Stool Mini Kit. The DNA was then eluted in 200 ​μl Buffer ATE. DNA was quantified using the Qubit™ 3.0 Fluorometer along with the high sensitivity DNA quantification assay kit.

The V3–V4 regions of the 16S rRNA gene are amplified and prepared for sequencing according to the 16S Metagenomic Sequencing Library Protocol. Two PCR reactions are performed on the extracted DNA. The DNA was first amplified using primers specific to the V3–V4 regions of the 16S rRNA gene: (Forward primer 5′TCGTCGGCAGCGTCAGATGTGTATAAGAGACAGCCTACGGGNGGCWGCAG; Reverse primer 5′GTCTCGTGGGCTCGGAGATGTGTATAAGAGACAGGACTACHVGGGTATCTAATCC). Each reaction contained 2.5 ​μl genomic DNA, 5 ​μl forward primer (1 ​μM), 5 ​μl reverse primer (1 ​μM) and 12.5 ​μl 2X Kapa HiFi Hotstart ReadyMix. PCR amplification was carried out using the following program: 95 ​°C ​× ​3 ​mins, 25 cycles of 95 ​°C ​× ​30 ​s, 55 ​°C ​× ​30 ​s, 72 ​°C ​× ​30 ​s, 72 ​°C ​× ​5 ​mins and held at 4 ​°C. PCR products were visualised using gel electrophoresis and then purified using AMPure XP beads. Following this, a second PCR reaction was carried out on the purified DNA using two indexing primers per sample. Each reaction contained 5 ​μl purified DNA, 5 ​μl index 1 primer (N7xx), 5 ​μl index 2 primer (S5xx), 25 ​μl 2x Kapa HiFi Hot Start Ready mix and 10 ​μl PCR grade water. The PCR amplification was completed using the previous program but with only 8 amplification cycles instead of 25. PCR products were visualised and purified as described above. Samples were quantified using the Qubit™ 3.0 Fluorometer along with the high sensitivity DNA quantification assay kit and then pooled in an equimolar fashion (20 ​nM). The sample pool was prepared following Illumina guidelines and sequenced on the MiSeq sequencing platform in Teagasc Moorepark, Fermoy using standard Illumina sequencing protocols.

#### 2.4.1 Bioinformatic sequence analysis

Three hundred base pair paired-end reads were assembled using FLASH (FLASH: fast length adjustment of short reads to improve genome assemblies). Further processing of paired-end reads including quality filtering based on a quality score of >25 and removal of mismatched barcodes and sequences below length thresholds was completed using QIIME. Denoising, chimera detection and clustering into operational taxonomic units (OTUs) (97% identity) were performed using USEARCH v7 (64-bit) (Edgar, 2010). OTUs were aligned using PyNAST (PyNAST: python nearest alignment space termination; a flexible tool for aligning sequences to a template alignment) and taxonomy was assigned using BLAST against the SILVA SSURef database release v123. Alpha and beta diversities were generated in QIIME (Caporaso et al., 2010) and calculated based on weighted and unweighted Unifrac distance matrices.

### 2.5 Fresh faecal plating

Samples were processed on arrival to the study laboratory. Culture based analysis was performed on the stool samples. Fresh faecal samples were weighed and serially diluted in maximum recovery diluent (Fluka, Sigma Aldrich, Ireland) from 10^−1^ to 10^−8^. Bifidobacteria were enumerated by spread-plating serial dilutions onto de Man, Rogosa, Sharpe (MRS) agar (Difco, Becton-Dickenson Ltd., Ireland), which had been modified by adding 0.05% L cysteine hydrochloride (Sigma Aldrich, Ireland), 100 μg/ml mupirocin (Sigma Aldrich, Ireland) and 50 units of nystatin (Sigma Aldrich, Ireland). Agar plates were incubated anaerobically for three days at 37 ​°C. *Lactobacillus* selective (LBS) agar (Difco, Becton-Dickenson Ltd., Ireland), supplemented with 50 units of nystatin was used to enumerate lactobacilli. Agar plates were incubated anaerobically for five days at 37 ​°C. Total anaerobic bacteria were enumerated by spread plating onto Wilkins Chalgren agar (WCA) (Sigma Aldrich, Ireland) supplemented with 50 units of nystatin and 7% defibrinated horse blood (Cruinn Diagnostics Ltd., Ireland). Agar plates were then incubated anaerobically for five days at 37 ​°C. Brain Heart Infusion (BHI) agar supplemented with 50 units of nystatin was used to enumerate total aerobic bacteria. These were also incubated anaerobically for five days at 37 ​°C.

### 2.6 Statistical analysis

The analyses were done on the intention-to-treat population. Dependent sample t-tests were used to explore differences between groups regarding days on treatment, compliance, and the PASA. To allow for repeated measures analysis and to avoid bias that may be introduced by using list-wise deletion of incomplete cases (Graham, 2009), missing data analysis was performed on physiological, psychological and cognitive variables subject to repeated measures analysis. In total, 1.03% of data was missing and determined to be missing completely at random (MCAR) using Littles MCAR test (Little, 1988); χ (3492) ​= ​228.95, p ​= ​1.00. Missing values were input by assigning the group mean for that variable except for cortisol awakening response data. All analyses were performed with missing data excluded (data not shown) and missing data included, which showed that inputting values using this method did not significantly change the nature of the results. Following missing data insertion, normality checks were performed using the Shapiro-Wilk test and visual inspection of histograms. Outliers were checked using box and whisker plots and only extreme outliers were considered for exclusion from analysis. PASA (challenge) and CANTAB RVP (total hits, total hits block 1 to 7) data was transformed using a reflect logarithm (LG10) transformation. CANTAB ERT (anger chosen, disgust chosen, surprise chosen), CANTAB RVP (mean latency, median latency, total misses, total misses block 1 to 7), GSR, and FFQ (E, H) data was not normally distributed and transformed using a natural log transformation (ln); GI-VAS data (satisfaction) data was transformed using a square-root transformation; PSQI, delta of PSQI (sleep duration), IPAQ, Reading the mind in the eyes, CANTAB MOT (mean error), CANTAB ERT (percentage correct, total number correct), CANTAB SSP (span length, number of attempts span 8, total usage errors, mean time to last response span 8), CANTAB PAL, CANTAB RVP (probability of hit, probability of false alarms, total false alarms, total correct rejections), LCC, GI-VAS (life interference), BDI-II, PSS, and FFQ (C) data was not normally distributed, but no transformations improved normality, so non-parametric analyses were used. Salivary cortisol awakening response values at each time-point were converted to area under the curve with respect to ground (AUCg) values (Pruessner et al., 2003). AUCg cortisol data was not normally distributed and no transformations improved normality, so again non-parametric analyses were used. PASA (stress index, challenge, self-concept of own abilities, control expectancy), CANTAB MOT (mean latency, median latency), CANTAB SSP (total errors), CANTAB ERT (happiness chosen, sadness chosen, fear chosen), and FFQ (A, B, D, F, G, I, J) were normally distributed and no transformations were performed. Following data imputation and transformation (if needed) to improve normality, repeated measures analysis of variance (ANOVA) with Time and Treatment as the within-subject factors for each variable was performed. Significant interaction effects were followed by post -hoc comparisons with paired sample t-tests using a Benjamini-Hochberg (BH) correction with a false discovery rate (FDR) of 0.10 for multiple comparisons as appropriate. Non-parametric equivalents, Friedman and Wilcoxon respectively, were used if parametric assumptions were violated. Data in table are presented as mean ​± ​SEM or %. P-Values <0.05 were considered statistically significant. Partial eta-squared (η^2^) was used to estimate effect size. Effects sizes were interpreted as following: η^2^ ​≤ ​0.06 was considered small, 0.06 > η^2^ ​≤ ​0.14 was considered moderate, η^2^ ​≥ ​0.14 was considered large. An α of 0.05 was considered significant. GraphPad Prism 7 was used to create graphs.

## 3 Results

### 3.1 Study participant profile

Thirty participants were enrolled and randomised with a total of 20 males completing the study with an average age of 20.7 (±0.28) years of age (Table 1). Baseline psychological measurements (Table 3) along with clinical measurements (data not shown) and Intelligence Quotient (IQ) were all considered within normal ranges. Comparing baseline and post-intervention measurements (Table 3), compliance was comparable across both groups. Similarly, body mass index (BMI) and the length of time on each treatment was equivalent across both groups. All participants in the study completed the GI-VAS, (Table 4, **Methods, 2.3.11**); a patient-reported questionnaire measuring abdominal pain, bloating, satisfaction and whether treatment interfered with their day to day lives which showed comparable scores at both baseline and following treatment across both groups. Furthermore, nutritional intake was similar across both groups pre and post intervention (Supplementary Table 1), while alcohol intake was significantly increased following probiotic treatment in semester 2 and physical activity decreased significantly for the placebo group during the exam stress period of the study (p ​< ​0.002).

**Table 1:** Patient demographics.

Population Characteristics	Total Sample (N ​= ​20)
Age (years, s.e.m)	20.7 (0.28)
Mode of delivery (N, %)	
• Vaginal	19 (95)
• Caesarean section	1 (5)
Ethnicity (N, %)	
• Caucasian	18 (90)
• Asian	1 (5)
• Middle Eastern	1 (5)
Units of alcohol per week (s.e.m)	8.94 (1.59)
Years of education (s.e.m)	15.63 (0.38)
IQ∗ (s.e.m)	108.41 (1.51)

s.e.m (standard error of the mean), IQ (intelligence quotient).

∗N ​= ​17.

**Table 2:** Outline of visit procedures.

Procedure	Visit 1 Screen	Visit 2 Baseline 1	Intervention Phase 1	Visit 3 Phase 1 End	Visit 4	Visit 5 Baseline 2	Intervention Phase 2	Visit 6 Phase 2 End
**Informed consent**	X							
**Inclusion/exclusion**	X							
**General medical history**	X							
**Demographic data**	X							
**MINI International Psychiatric Interview**	X							
**Self-report scales** (Childhood Trauma Questionnaire-Short Form/TIPI/ROME III/Cambridge Behaviour Scale/Interpersonal Reactivity Index/Autism Quotient/Handedness/WAYS)	X							
**Cognitive Assessment** (NART)	X							
**Physical exam**		X		X		X		X
Heart rate, heart rate variability & galvanic skin response		X		X		X		X
**Self-report scales** (Hopkins Symptom Checker/Hospital Anxiety and Depression Scale/Beck Depression Inventory-II/State-Trait Anxiety Inventory/Perceived Stress Scale/Pittsburgh Sleep Quality Index/International Physical Activity Questionnaire /GI symptoms VAS/Bristol Stool Chart)		X		X		X		X
**Food Frequency Questionnaire**	X			X				X
**Cognitive Assessment** (CANTAB/Reading the Eyes in The Mind Test)	X (Practice)	X		X	X (Practise)	X		X
**Intervention** (probiotic or placebo)			X				X	
**Self-Report Scales during Intervention** (Perceived Stress Scale, Positive and Negative Affect Schedule, GI symptoms VAS)			X				X	
**Exam stress**				X				X
**Self-Report Scales During Exam stress** (Primary Appraisal Secondary Appraisal/Positive and Negative Affect Schedule/Bond-Lader visual analogue mood rating scales)				X				X
**Stool sample**		X		X		X		X
**Hair sample**		X		X		X		X
**Saliva sample** (x8 for cortisol awakening response collected in 2 consecutive mornings (4 samples each morning))		X		X				X
**Adverse events**		X	X	X		X	X	X
**Concomitant Medications Record**	X	X		X	X	X		X
**Intervention Palatability**				X				X

**Table 3:** Baseline measurements.

Baseline psychological measurements	Total Sample (N ​= ​20)
CTQ score (SE)	
• Emotional abuse	6.45 (1.54)
• Physical abuse	5.70 (1.42)
• Sexual abuse	5.00 (0.00)
• Emotional neglect	6.95 (1.91)
• Physical neglect	5.30 (0.57)
TIPI Score (SE)	
• Extraversion	4.75 (1.67)
• Agreeableness	5.15 (1.08)
• Conscientiousness	5.05 (1.29)
• Emotional stability	5.78 (1.18)
• Openness to experience	5.28 (0.95)
CBS score (SE)	47.80 (1.66)
IRI score (SE)	
• Perspective taking	21.65 (0.92)
• Fantasy	17.75 (1.15)
• Empathic concern	20.50 (1.16)
• Personal distress	8.40 (0.80)
AQ score (SE)	12.85 (1.31)
STAI trait anxiety score (SE)	31.10 (1.40)
WAYS score (SE)	
• Confrontive coping	6.15 (0.64)
• Distancing	6.80 (0.43)
• Self-controlling	10.90 (0.81)
• Seeking social support	8.50 (0.80)
• Accepting responsibility	5.00 (0.64)
• Escape-avoidance	5.45 (0.82)
• Planful problem-solving	10.05 (0.71)
• Positive reappraisal	6.95 (0.78)

Values are the mean score (SEM) or frequency (%). IQ: Intelligence Quotient; CTQ: Childhood Trauma Questionnaire; TIPI: Ten Item Personality Inventory; CBS: Cambridge Behaviour Scale; IRI: Interpersonal Reactivity Index; AQ: Autism Quotient; STAI: State Trait Anxiety Inventory; WAYS: Ways of Coping Questionnaire.

**Table 4:** Participant metadata pre- and post-measurement.

	Placebo baseline (N ​= ​20)	Placebo exam stress (N ​= ​20)	p	Probiotic baseline (N ​= ​20)	Probiotic exam stress (N ​= ​20)	p
Compliance	–	102.5	–	–	94.75	ns
Days on treatment	–	52.55	–	–	53.2	ns
BMI	24.14 (0.65)	24.26 (0.72)	ns	24.23 (0.77)	24.1 (0.71)	ns
MET-min/week	5805 (3689)	3408 (2895)	0.002	4529 (2973)	4111 (2903)	ns
**GI-VAS**						
• Abdominal pain (%)	0	1 (5)	ns	0	0	
• Bloating (%)	0	1 (5)	ns	1 (5)	2 (10)	
• Satisfaction	25.14 (4.83)	17.04 (4.67)		20.35 (5.09)	22.76 (5.52)	ns
• Life interference	11.54 (2.93)	8.56 (2.91)		9.35 (2.88)	9.75 (2.94)	ns
Bristol Stool Chart	3.33 (1.34)	3.88 (1.51)	ns	3.32 (1.56)	3.50 (1.24)	ns

Values are the mean score ​± ​S.E.M or frequency (%). Physical activity, expressed in MET-minutes per week, decreased significantly for placebo (p ​= ​0.002). BMI: Body Mass Index; MET: metabolic equivalent; GI-VAS: Gastrointestinal Visual Analogue Scale. Ns ​= ​not significant.

### 3.2 Effect of exam period, but not of probiotic, on psychological markers of stress

**Fig. 2:**
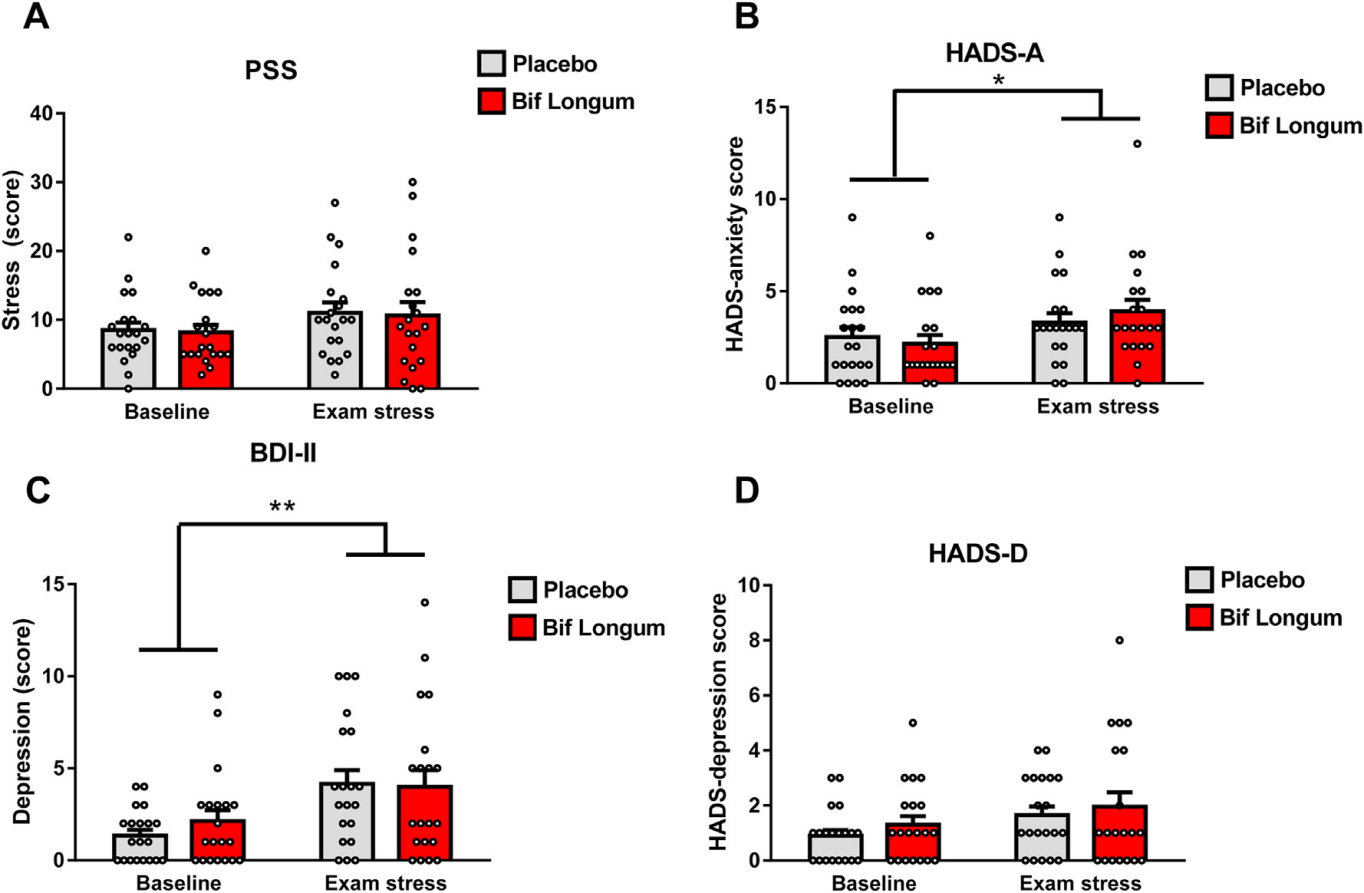
**Chronic exam stress increases self-reported measures of anxiety**. (A) The Perceived Stress Scale (PSS) questionnaire was taken before and during the exam period in each participant. **(B, D)** The Hospital anxiety and disease scale (HADS-A, HADS-D) was self-reported before (baseline) and during exam stress. The Beck’s Depression Inventory second edition (BDI-II) was self-reported by participants before and during exam the exam period **(C).** Data represented in bar graphs with grey corresponding to the placebo group and red corresponding to participants treated with *Bif. longum* with individual white dots indicating individual data points, data is presented as averages and error bars represent the standard error of the mean s.e.m, n ​= ​8 in 1714 and n ​= ​12 in placebo group. ∗p ​= ​0.05, ∗∗p ​= ​0.01.(For interpretation of the references to colour in this figure legend, the reader is referred to the web version of this article.)

To investigate the possibility for *B. longum* 1714 (*Bif. longum*) supplementation to positively enhance stress, mood, memory and cognitive ability we utilised the naturalistic stressor of the university exam period as our chronic stress paradigm. Overall, the students self-reported that exams in semester 1 and semester 2 were equally as difficult and stressful (data not shown), thus we do not make the distinction between semesters in our analysis. To confirm participant’s baseline stress levels at the start of the study (term-time), several self-reported questionnaires were filled out by the participants. At baseline, the perceived stress score (PSS) was not significantly different between placebo and 1714 (t = (18) −0.901, *p* ​= ​0.381), and the average score was less than 13 which would indicate a low level of stress (Fig. 2A), (Cohen et al., 1983). Subsequently, as expected, following exam stress, the PSS scores increased, but there was no difference in the PSS score between groups receiving placebo (F (1,17) ​= ​0.003, *p* ​= ​0.961, η^2^ ​= ​0) or *Bif. longum* (F (1,17) ​= ​0.007, *p* ​= ​0.932, η^2^ ​= ​0) after controlling for the effect of baseline scores, indicating that both groups responded to exams similarly, with no effect of the probiotic. To tease apart the potential influence of anxiety and depression on our study participants and using the HADS questionnaire we found that anxiety increased significantly in both *Bif. longum* and placebo groups (Fig. 2B) while *Bif. longum* did not have any effect on reported anxiety compared to placebo. Similarly, self-reported depression scores increased in semester 2 in both placebo and *Bif. longum* groups (not significantly) while like HADS-A, there was no difference in HADS-D scores between placebo and *Bif. longum* groups (Fig. 2C).

Further validation of the psychometric status of our participants came from self-reported measures using the BDI-II questionnaire (Fig. 2D). Like the HADS-A and HADS-D measures, baseline BDI-II scores were not significantly different at baseline (t = (18) −1.274, *p* ​= ​0.219) and scores increased significantly during the exam season but did not differ between treatment groups controlling for baseline scores (placebo, F(1,17) ​= ​3.946, *p* ​= ​0.06, η2 ​= ​0.18, *Bif. longum*, F (1,17) ​= ​0.318, *p* ​= ​0.58, η2 ​= ​0.018) confirming that *Bif. longum* had no effect on self-reported anxiety or depression in chronically stressed students. To further classify the stress phenotype of our patient cohort we psychometrically evaluated all patients using a cognitive appraisal questionnaire, the PASA, at the post-intervention visit (Fig. 1A, Table 5). When we evaluated the 4 main cognitive appraisal processes (both primary and secondary) “threat”, (t = (18) −1.672, *p* ​= ​0.112), “challenge”, (t = (18) −1.309, *p* ​= ​0.207), “self-concept of own abilities”, (t = (18) 0.772, *p* ​= ​0.450), and “control expectancy”, (t = (18) 0.537, *p* ​= ​0.598), we found no difference in any of the sub-categories or in the cumulative score confirming that our participants, regardless of treatment group, anticipated stress and anxiety due to the exam period in a similar manner. Thus, our primary objective, to reduce chronic stress during an exam period using the probiotic *Bif. longum* was ineffective.

**Table 5:** Primary appraisal, secondary appraisal scores.

	Placebo	Bif Longum	*t* (18)	*p*
Stress Index	64 ​± ​14.31	65.10 ​± ​14.56	−0.77	0.230
Threat	13.35 ​± ​2.99	13.65 ​± ​3.05	−1.672	0.122
Challenge	20.9 ​± ​4.67	20.8 ​± ​4.65	−1.309	0.207
Self-concept of own abilities	10.25 ​± ​2.29	10.6 ​± ​2.37	0.772	0.450
Control Expectancy	19.5 ​± ​4.36	20.05 ​± ​4.48	0.537	0.600

Data is presented as the mean ​+ ​the standard error of the mean (SEM).

### 3.3 Cognitive assessment

While we had successfully established a stable baseline phenotype in our subjects and previous work from our group had shown *Bif. longum* to be effective at improving neurocognitive performance following acute stress, we wanted to assess its effect on cognitive performance in a chronic stress setting. Using a selection of cognitive tests from the CANTAB battery (Table 6), we measured visual memory and learning **(PAL)**, sustained attention **(RVP)**, working memory **(SSP)**, emotional recognition **(ERT)** and social cognition **(RMIE)**. At baseline, there was a significant difference in the RVP mean latency (Z ​= ​−2.053, *p* ​= ​0.04) there was no significant difference between subjects receiving placebo or *Bif. longum* when assessing PAL, total errors adjusted (Z ​= ​−1.530, *p* ​= ​0.132), PAL total errors adjusted 8 shapes (Z ​= ​0.756, *p* ​= ​0.470), PAL mean trials to success (Z ​= ​−1.180, *p* ​= ​0.257), RVP total hits (Z ​= ​- 0.222, *p* ​= ​0.836), RVP total misses (Z ​= ​- 0.222, *p* ​= ​0.836), SSP span length (Z ​= ​- 0.247, *p* ​= ​0.0873), ERT correct responses (Z ​= ​−0.206, *p* ​= ​0.848) and Reading the mind in the eyes (Z ​= ​- 1.003, *p* ​= ​0.329). When assessing if treatment with placebo or *Bif. longum* was effective in improving visual memory using the PAL test, there was no difference in the total number of errors (placebo, F (1,17 ​= ​0.125, p ​= ​0.728, η2 ​= ​0.007), *Bif. longum*, F (1,17 ​= ​1.570, *p* ​= ​0.227, η2 ​= ​0.085), total errors adjusted for 8 shapes (placebo, F (1,17 ​= ​0.028, *p* ​= ​869, η2 ​= ​0.002, *Bif. longum*, F (1,17, ​= ​1.373, *p* ​= ​0.258, η2 ​= ​0.075) or in the number of trials to success (placebo, F (1,17 ​= ​0.239, *p* ​= ​0.631, η2 ​= ​0.014, *Bif. longum*, F (1,17 ​= ​1.66, *p* ​= ​0.215, η2 ​= ​0.089) controlling for the covariate of baseline scores. In the sustained attention task (RVP), there was a significant increase in the number of correct hits in both the placebo (Z ​= ​2.395, *p* ​= ​0.017) and *Bif. longum* (Z ​= ​−3.468, *p* ​= ​0.001) groups during exams stress period compared with baseline, although no difference in correct hits was noted between placebo (F(1,17, ​= ​0.434, p ​= ​0.519, η2 ​= ​0.025) and *Bif. longum* (F (1,17 ​= ​0.465, p ​= ​0.504, η2 ​= ​0.027) during the exam stress period. Similarly, when examining the number of misses in the RVP test, a significant decrease in the number of incorrect selections were noted in placebo, (Z ​= ​2.395, *p* ​= ​0.017) and *Bif. longum*, (Z ​= ​−3.468, *p* ​= ​0.001) during the exam stress period, this decrease did not differ significantly between placebo F (1,17, ​= ​0.434, p ​= ​0.519, η2 ​= ​0.025) and *Bif. longum* (F (1,17 ​= ​0.465, p ​= ​0.504, η2 ​= ​0.027) during exam stress (Fig. 5E). Finally, there was no significant change in the latency to respond during the exam stress period or between placebo (F1,17 ​= ​1.664, *p* ​= ​0.214, n2 ​= ​0.089) and *Bif. longum* (F1,17, ​= ​1.375, *p* ​= ​0.257, n2 ​= ​0.075) controlling for baseline scores.

**Table 6:** Cognitive assessment.

CANTAB Test (unit of measurement)	Intervention	Study Period	Mean ​± ​SEM	Baseline Comparison∗	Exam Stress^$^	Placebo v 1714^%^
PAL Total Errors adjusted	Placebo	Baseline	3.80 ​± ​0.70	NS		
(no. of errors)		Exam Stress	2.42 ​± ​0.54		NS	NS
	B. longum	Baseline	2.26 ​± ​0.58			
		Exam Stress	2.30 ​± ​0.50		NS	
PAL total errors adjusted 8	Placebo	Baseline	2.00 ​± ​0.54	NS		
(no. of errors)		Exam stress	2.00 ​± ​0.46		NS	NS
	B. longum	Baseline	1.37 ​± ​0.39			
		Exam stress	1.75 ​± ​0.42		NS	
PAL Mean Trials to success	Placebo	Baseline	1.32 ​± ​0.05	NS		NS
(no. of trials)		Exam Stress	1.26 ​± ​0.06		NS	
	B. longum	Baseline	1.22 ​± ​0.05			
		Exam Stress	1.22 ​± ​0.05		NS	
RVP Total Hits	Placebo	Baseline	21.40 ​± ​0.94	NS		NS
(no. of hits)		Exam Stress	23.30 ​± ​0.67		∗∗	
	B. longum	Baseline	21.21 ​± ​0.79			
		Exam Stress	23.80 ​± ​0.68		∗∗∗	
RVP Total Misses	Placebo	Baseline	5.60 ​± ​0.94	NS		NS
(no. of misses)		Exam stress	3.70 ​± ​0.67		∗∗	
	B. longum	Baseline	5.79 ​± ​0.79			
		Exam stress	3.20 ​± ​0.68		∗∗∗	
RVP Mean Latency	Placebo	Baseline	384.56 ​± ​15.63	∗		NS
(milliseconds)		Exam stress	356.81 ​± ​10.12		NS	
	B. longum	Baseline	360.79 ​± ​11.20			
		Exam stress	355.26 ​± ​12.08		NS	
SSP Span Length	Placebo	Baseline	7.65 ​± ​0.18	NS		NS
(no. of stimuli recalled)		Exam stress	8.30 ​± ​0.22		∗∗	∗
	B. longum	Baseline	7.60 ​± ​0.18			
		Exam Stress	8.50 ​± ​0.20		∗∗	
ERV Percent Correct	Placebo	Baseline	75.97 ​± ​0.98	NS		NS
(percentage)		Exam stress	77.56 ​± ​1.02		NS	
	B. longum	Baseline	76.03 ​± ​1.15			
		Exam stress	78.19 ​± ​1.03		NS	
Reading the Mind in the Eyes	Placebo	Baseline	27.05 ​± ​0.70	NS		
(score)		Exam stress	28.05 ​± ​0.73		NS	∗
	B. longum	Baseline	27.55 ​± ​0.66			
		Exam stress	27.65 ​± ​0.73		NS	

NS ​= ​not significant, ∗baseline comparison at visit 1 pre-treatment, ^$^baseline versus exam stress, ^%^placebo versus probiotic during exam period.

When we examined working memory using the spatial span test (SSP) there was a significant effect of exam stress on the number of stimuli recalled for both placebo (Z ​= ​−2.415, *p* ​= ​0.016) and *Bif. longum* (Z ​= ​−2.717, *p* ​= ​0.007) and a significant difference between placebo (F1,17 ​= ​6.693, *p* ​= ​0.019, n2 ​= ​0.282) and *Bif. longum* (F1,17 ​= ​0.123, *p* ​= ​0.73, n2 ​= ​0.007) during exam stress controlling for baseline scores. Furthermore, when evaluating emotional recognition using the ERT, no difference was observed in the percentage of correct responses at baseline or during the exam period, in addition, no differences were noted between placebo (placebo, F(1,17 ​= ​0.24 *p* ​= ​0.63, n2 ​= ​0.014) or *Bif. longum* (F (1,17 ​= ​0, *p* ​= ​0.991, n2 ​= ​0) groups during the exam stress period. Finally, when subjects were assessed on their ability to attribute mental states to others using the Reading the Mind in the Eyes test, an effect of exam stress was noted in placebo F(1,17 ​= ​6.226, *p* ​= ​0.025, n2 0.262) and no difference was noted in *Bif. longum* F (1,17 ​= ​0.008, *p* ​= ​0.928, n2 ​= ​0).

### 3.4 Chronic stress evaluation

**Fig. 3:**
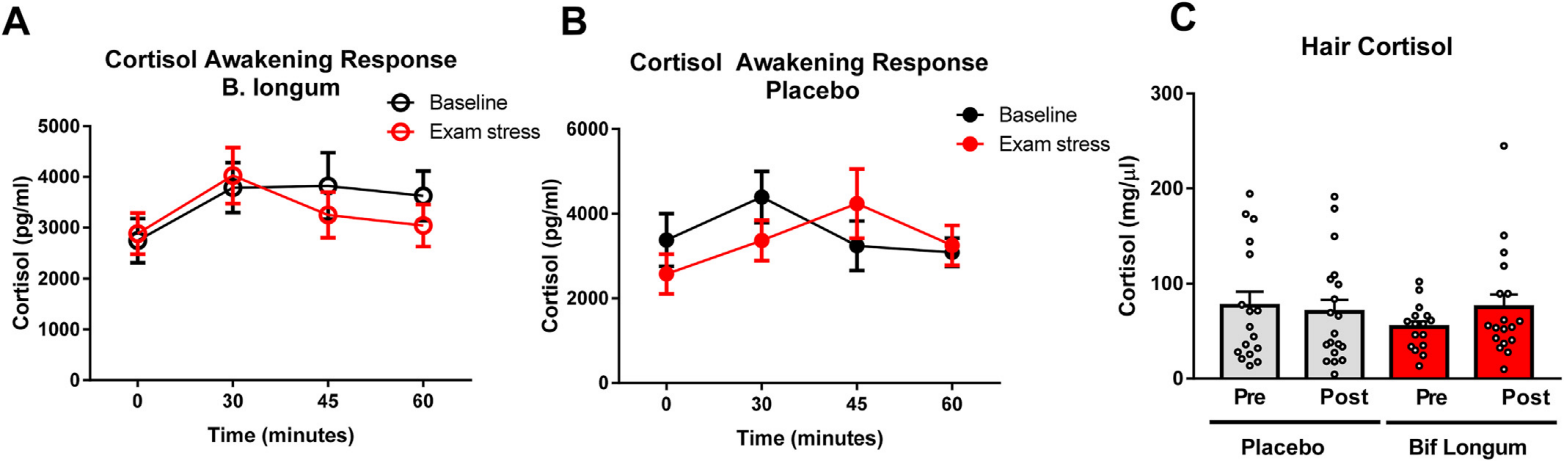
***B. longum* does not improve Cortisol awakening response during chronic exam stress** The salivary Cortisol awakening response (CAR) was measured upon wakening in the morning at time 0 and at 15-min intervals from this point on 3 occasions to give a total of 4 samples for placebo and probiotic before and during chronic exam stress. Samples were measured by ELISA and no significant differences were noted in the placebo **(A)**, probiotic, *Bif. longum***(B)** and in the area under the curve (AUC), **(C)**. Before and during exam stress, hair samples were analysed for cortisol levels by ELISA, no significant differences were noted between groups **(D)**. **(A**–**B)**, Data represented as scatter plots, data represented by averages and error bars represent the standard error of the mean s.e.m Data represented as bar-plots **(C**–**D)** with individual data points represented by white dots, placebo in grey, probiotic in red. Data represented by averages and error bars represent the standard error of the mean s.e.m, n ​= ​8 in *Bif. longum* and n ​= ​12 in placebo group. (For interpretation of the references to colour in this figure legend, the reader is referred to the web version of this article.)

Previous work from our group had shown efficacy of *Bif. longum* in reducing hypothalamic-pituitary-adrenal axis activity, specifically salivary cortisol in healthy volunteers following an acute stressor, the socially evaluated cold pressor test (SECPT), (Allen et al., 2016b). To evaluate the capacity of *Bif. longum* to reduce cortisol levels in saliva during a period of chronic stress we measured cortisol before and during exam periods at time 0 (awakening) and at 15-min intervals thereafter up until 60 ​min post awakening (Fig. 1, Fig. 3A and B). At the first study visit, baseline salivary cortisol levels were not significantly different (Z ​= ​−0.966, *p* ​= ​0.352). Using a repeated measures ANOVA, there was no significant change in salivary cortisol at any timepoint in placebo (Fig. 3A, Wilk’s lambda ​= ​0.870, F = (3,17) 0.844, *p* ​= ​0.489, n2 ​= ​0.130) and *Bif. longum* (Fig. 3B, Wilk’s lambda ​= ​0.510, F = (3,17) 1.601, *p* ​= ​0.301, n2 ​= ​0.490), before treatment. Following treatment, there was no effect of placebo (Fig. 3A, Wilk’s lambda ​= ​0.792, F = (3,17) 1.490, *p* ​= ​0.253, n2 ​= ​0.208) or *Bif. longum* (Fig. 3B, Wilk’s lambda ​= ​0.830, F = (3,17) 1.158, *p* ​= ​0.355, n2 ​= ​0.170) on salivary cortisol output. Measuring the relative change in area under the curve there was no significant change in salivary cortisol output following treatment with placebo (Fig. 3C, F (1,16, ​= ​0.109, *p* ​= ​0.746, n2 ​= ​0.007)) or *Bif. longum* (F (1,16, ​= ​2.160, *p* ​= ​0.161, n2 ​= ​0.119) when controlling for cortisol levels at the baseline visit. Of note, the increase in cortisol output at the first timepoint (30 ​min after waking up) was not statistically significant in placebo (Z ​= ​−1.680, *p* ​= ​0.097) or *Bif. longum* (z ​= ​- 0.971, *p* ​= ​0.083) but a tendency to increased cortisol production was observed. A more retrospective measurement of cortisol output and HPA activity was carried out using hair from each participant. There was no difference in hair cortisol levels between participants receiving placebo or *Bif. longum* at baseline (Fig. 3D, Z ​= ​- 0.104, *p* ​= ​0.932). When controlling for baseline hair cortisol measurements there was no effect of placebo (Fig. 3D, F (1,32) ​= ​0.186, *p* ​= ​0.669, n2 ​= ​0.006) or *Bif. longum* (Fig. 3D, F (1,32) ​= ​0.620, *p* ​= ​0.444. n2 ​= ​0.042)

### 3.5 Sleep quality assessment

**Fig. 4:**
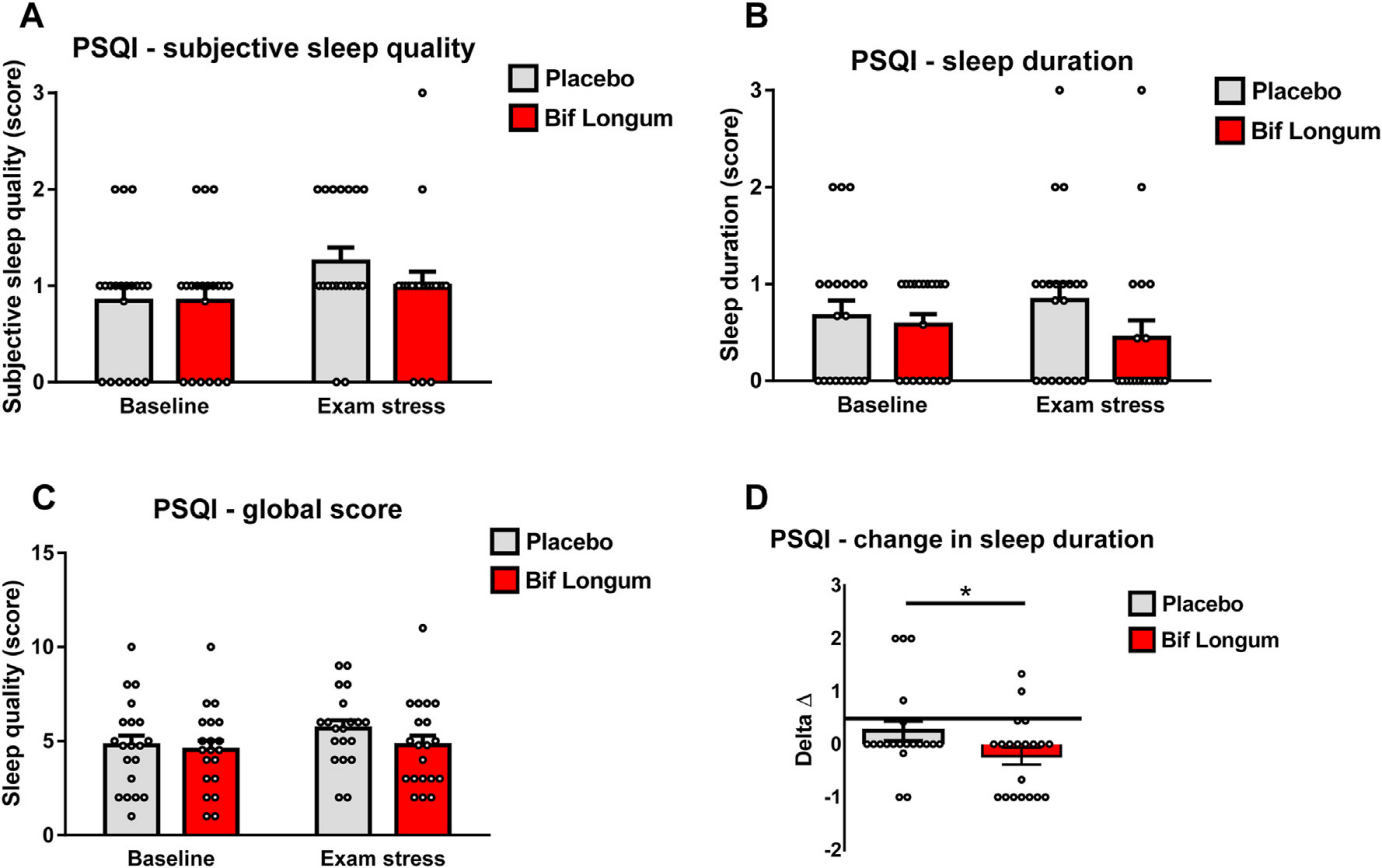
***B. longum* supplementation improves the duration of sleep during chronic stress**. Using the Pittsburgh sleep quality index (PSQI), the sleep quality score **(A)**, the duration of sleep **(B)** and the global sleep quality score **(C)** are represented by bar graphs grey bars representing participants receiving placebo and red bars representing participants receiving probiotic and white circles representing individual data points, with error bars representing the s.e.m, participants. The change in sleep duration is represented by graphs of alpha-diversity represented by box-whisker plots with data represented as median with inter-quartile range and min/max values as error bars **(D**, ∗p ​= ​0.05).(For interpretation of the references to colour in this figure legend, the reader is referred to the web version of this article.)

Prolonged periods of chronic stress can result in the nervous system maintaining a heightened state of arousal which can affect several physiological processes (Mcewen, 2017). Subjective sleep quality was assessed using the PSQI and at baseline there was no significant differences in subjective sleep quality (Fig. 4A, Z ​= ​−1.473, *p* ​= ​1), sleep duration (Fig. 4B, Z ​= ​0.522, *p* ​= ​0.648), PSQI global score (Fig. 4C, Z ​= ​−0.707, *p* ​= ​0.631), (2.3.15). When controlling for baseline scores, participants receiving *Bif.* *longum* had significantly improved sleep when compared to those receiving placebo. However, the positive change in sleep quality experienced by participants during the exam period was significantly improved when they consumed *Bif. longum* compared to those receiving placebo (Fig. 4D, Z ​= ​−2.068, p ​= ​0.039). This data suggests that *Bif. longum* may hold promise as a probiotic supplement that could improve sleep quality during periods of chronic stress such as exams.

**Fig. 5:**
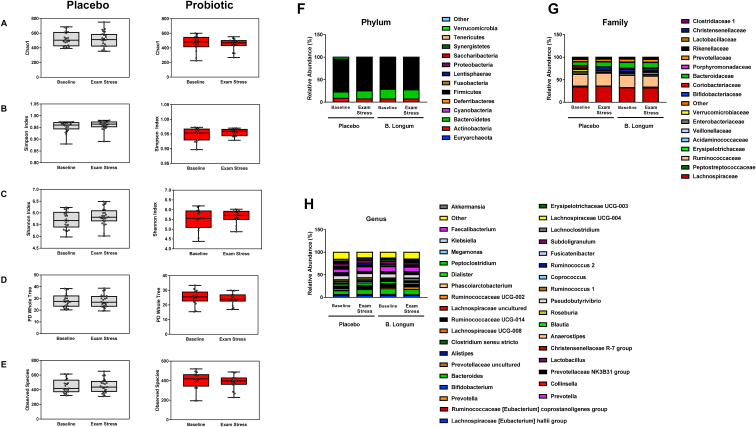
***B. longum* supplementation does not alter the faecal microbiome during exam stress.** Following 16S compositional sequencing of faecal samples from timepoints before and after each visit (Fig. 1A, Table 2) was performed. Species diversity was not changed significantly at any visit compared within or between placebo and probiotic groups as measured by Chao1 **(A)**, Simpson Index **(B)** Shannon Index **(C)** PD Whole Tree **(D)** and Observed Species **(E)**. Relative abundance at the phylum **(F)**, Family **(G)** and Genus **(H)** level there was no significant difference in the percentage of taxa in each group before or during the exam stress between participants receiving placebo or probiotic. Fig. 5 A-E, graphs of alpha-diversity represented by box-whisker plots with data represented as median with inter-quartile range and min/max values as error bars, n ​= ​8 in *Bif. longum* and n ​= ​12 in placebo group.

### 3.6 Chronic stress and the microbiome

Using 16S sequencing we assessed the effect of chronic stress on the microbiome and how specifically *Bif. longum* may modify the microbiome. When we assessed species diversity using various measures of alpha diversity, we found no effect of placebo or probiotic intervention on the Chao1 (Fig. 5A, placebo Z ​= ​1.725, *p* ​= ​1, probiotic Z ​= ​1.725, *p* ​= ​1), Simpson (Fig. 5B, placebo Z ​= ​1.725, *p* ​= ​1, probiotic Z ​= ​1.725, *p* ​= ​1) or Shannon (Fig. 5C, placebo Z ​= ​1.725, p ​= ​1, probiotic Z ​= ​1.725, p ​= ​1) index as well as the PD-whole tree (Fig. 5D, placebo Z ​= ​1.725, *p* ​= ​1, probiotic Z ​= ​1.725, *p* ​= ​1) and the number of observed species, (Fig. 5E, placebo Z ​= ​1.333, *p* ​= ​1, probiotic Z ​= ​1.333, *p* ​= ​1). At the phylum level, the microbiome profile at visit 1 and visit 2 in both groups was dominated by *Firmicutes* (71%) and *Bacteroidetes* (18%) before and during the exam period. The quantity of *Firmicutes* (placebo, Z ​= ​1.726, *p* ​= ​1, probiotic, Z ​= ​1.726, *p* ​= ​1) and *Bacteroidetes* (placebo, Z ​= ​1.726, *p* ​= ​1, probiotic, Z ​= ​1.726, *p* ​= ​1), (or any phyla) was not significantly affected by *Bif. longum* or placebo (Fig. 5F). Similarly, at the family level, no increase in abundance was noted before or after exams with the *Lachnospiraceae*, *Ruminococcaceae* and *Bacteroidaceae* families forming the most abundant in both groups. Equally, *Bif. longum* or placebo treatment had no effect on relative abundance at the family level (Fig. 5G). At the genus level, no genera were significantly changed between visits or by supplementation with *Bif. longum* or placebo, while *Bacteroides* (11%) and *Faecalibacterium* (10%) were the most abundant genera (Fig. 5H). We also examined fresh plated faeces from each participant before and after each semester (Fig. 1A), data not shown. There was no significant difference in plate counts for Total anaerobes (F (7, 78) ​= ​1.171 *p* ​= ​0.3291, Bifidobacteria F (7, 79) ​= ​0.7955 *p* ​= ​0.5933, Lactobacilli F (7, 79) ​= ​0.7146 *p* ​= ​0.6598 and Total aerobes F (7, 73) ​= ​0.8884 *p* ​= ​0.5202 between groups receiving placebo or probiotic before or during their exams.

## 4 Discussion

Several pre-clinical studies have suggested a potential role for probiotics in the treatment of stress and anxiety related disorders that have the potential to become clinically relevant psychobiotics (Dinan et al., 2013; Sarkar et al., 2018; Liu et al., 2018). Using a repeated measures design to control for individual variation we selected stress and cognitive tests that would examine memory, sustained attention, and emotional processing. Over the course of the study we found that although *Bif. longum* failed to improve self-reported increase in stress and anxiety due to the exam period it did have a positive effect on sleep. In addition, the composition of the microbiome before and during exams was not altered by *Bif. longum* supplementation. Similarly, *Bif. longum*, which was well tolerated by participants, did not modulate any facet of cognitive performance assessed using the comprehensive CANTAB battery of tests.

Importantly, our participants developed a stressful phenotype during the exam period, they have increased self-reported scores of stress and anxiety during the exam period including perceived stress (PAS) and anxiety (HADS_A), similarly, BDS-II scores are increased during the exam period but were considered low with regards depression. Similarly, cortisol awakening response was increased in both placebo and *Bif. longum* but no difference was noted between the group receiving *Bif. longum* compared to placebo, while a moderate improvement in the change in sleep quality was noted in patients receiving *Bif. longum* during the exam period.

Of note, our study participants had low levels of anxiety and depression (Fig. 2, Fig. 3) and peripheral cortisol (Fig. 3) at the beginning of the study, moreover, they self-selected for a study that took place during their exams, suggesting they were a particularly resilient (Table 3, **Ways of Coping Questionnaire**) cohort. Our results represent the many hurdles associated with the development of psychobiotics for use in humans. In fact, several rodent studies have shown cognitive and anti-stress benefits of supplementation with the strain *Bif. longum* in healthy mice (Savignac et al., 2014, 2015). Studies have shown that in stress sensitive BALB/c mice, a *Bif. longum* strain enhanced cognitive performance, learning and memory along with modulating behaviours related to anxiety (Tian et al., 2020). Furthermore, recent data from our lab showed that in healthy volunteers, *Bif. longum* was able to attenuate the physiological and psychological reaction to an acute stressor, the cold pressor test (Allen et al., 2016a). In addition, self-reported psychological stress was reduced along with enhanced frontal midline electroencephalographic mobility following psychobiotic consumption. Moreover, contrary to these findings in healthy volunteers undergoing an acute stressor (Wang et al., 2019), *Bif. longum* shows no similar effects in healthy participants undergoing a naturalistic chronic stress during a three-week exam period using a randomised, placebo-controlled, repeated measures, cross-over intervention.

Stress and sleep are fundamentally linked and anxiety can lead to poor sleep quality and a reduced duration of sleep in patients with IBS and other anxiety disorders (Vandekerckhove and Cluydts, 2010; Kim et al., 2018; Ramsawh et al., 2009). In addition, there is a strong relationship between stress and academic performance with low pre-exam stress positively associated with better exam performance (Ahrberg et al., 2012). Of interest, we expected the quality of sleep experienced by our participants to decrease during the exam period, but this was not the case, sleep quality remained similar to sleep duration levels before the exam period in both placebo and *Bif. longum* treated participants. Notably, sleep duration was improved by *Bif. longum* during the exam period, suggesting that *Bif. longum* could be beneficial during exam periods and generally in disorders with heightened anxiety. In 2017, Takada et al., demonstrated that the *Lactobacillus casei* strain Shirota improved sleep quality during periods of increasing academic stress (Takada et al., 2017), while clinically, in patients with Chronic Fatigue Syndrome, 2 months of supplementation with the Shirota strain reduced anxiety (Rao et al., 2009). Conversely, a study from 2019 showed that treating participants with a synbiotic for 6 weeks had no effect on sleep quality or duration during different periods of the academic calendar (Marotta et al., 2019). This data suggests that the positive effects of probiotic strains may be strain specific and that further studies examining the interaction of probiotic strains with sleep architecture are warranted.

Results from other studies looking at the treatment of anxiety and stress with psychobiotics varies in terms of efficacy. For example, in 2004, using the mixed culture Actimel ®, Danone, France) containing the cultures *Lactobacillus delbrueckii bulgaricus* (10^7^/mL), *Streptococcus salivarius thermophilus* (10^8^/mL), and *Lactobacillus casei* DN-114001 (10^8^/mL), Marcos et al., in a randomised controlled, parallel, prospective design found no effect of supplementation with a mixed probiotic strain on anxiety traits, serum cortisol or peripheral markers of immune activation during an exam stressor (Marcos et al., 2004). In a randomised, double-blind, placebo-controlled study using Yakult™ for a 3-week period, healthy volunteers with a lower baseline mood experienced a reduction in depressed mood assessed using a VAS (Benton et al., 2007) but long-term memory was not affected. Our data, and that of Benton et al. tend to agree with the hypothesis that probiotics work better in patients with a lower baseline mood than those with an optimal baseline VAS score. Indeed, our previous pre-clinical data on the potential use of *Bif. longum* for reducing anxiety was carried out using BALB/c mice, an anxious inbred strain (Michalikova et al., 2010). Similarly, in 2017 we found no effect of the *Lactobacillus rhamnosus* strain on mood, anxiety, stress, and sleep quality in a cohort of healthy volunteers, once again suggesting that psychobiotics may be more effective in studies with moderate anxiety (Kelly et al., 2017; Colica et al., 2017; Slykerman et al., 2017; Papalini et al., 2019). Overall a recent meta-analysis suggests that utilising psychobiotics may be a potentially useful adjunctive treatment. Furthermore, patients with certain co-morbidities, such as irritable bowel syndrome might experience greater benefits from such treatments (Noonan et al., 2020)

Our study is not without limitations, these include the fact that our sample size was small, and our participants were healthy and volunteered for a study during their exam period. Overall, ten patients withdrew from the study with 7 of them being from our treatment group which reduced our statistical power (n=9 prior to commencement of the intervention). Indeed, it is possible that *Bif. Longum* would be more efficacious in conditions with an anxious phenotype such as irritable bowel syndrome or depression. Furthermore, we did not examine brain imaging or EEG which has shown promise as a functional readout of efficacy in probiotic strains (Pinto-Sanchez et al., 2017; Allen et al., 2016b).

The use of probiotics to target the gut microbiome in psychiatry and in specific, disorders of stress and anxiety holds much promise, the role for specific strains in specific clinical conditions requires more data and the data presented here is intended to add to this field. In a prolonged period of chronic stress, *Bif. longum* although failed to modify feelings of anxiety, decrease levels of stress or improve cognitive performance it had beneficial effects on sleep parameters. While further mechanistic research is warranted as to why the duration of sleep improves during chronic stress with *Bif. longum* supplementation, our data further supports the concept of probiotics modulating brain health.

## Declaration of competing interest

This publication has emanated from research supported, in part, by a research grant from Science Foundation Ireland (SFI) to APC Microbiome Ireland under grant SFI/12/RC/2273. The authors received additional funding from the European Community’s Seventh Framework Program Grant MyNewGut under grant FP7/2007–2013. The centre has conducted studies in collaboration with several companies, including GSK, Pfizer, Cremo, Suntory, Wyeth, and Mead Johnson.
